# Altered brain metabolism in frontotemporal dementia and psychiatric disorders: involvement of the anterior cingulate cortex

**DOI:** 10.1186/s13550-023-01020-2

**Published:** 2023-07-26

**Authors:** Marie-Paule E. van Engelen, Sander C. J. Verfaillie, Annemieke Dols, Mardien L. Oudega, Ronald Boellaard, Sandeep S. V. Golla, Marijke den Hollander, Rik Ossenkoppele, Philip Scheltens, Bart N. M. van Berckel, Yolande A. L. Pijnenburg, Everard G. B. Vijverberg

**Affiliations:** 1 Alzheimer Center Amsterdam, Neurology, Vrije Universiteit Amsterdam, Amsterdam UMC Location VUmc, Amsterdam, The Netherlands; 2 Amsterdam Neuroscience, Neurodegeneration, Amsterdam, The Netherlands Alzheimer Center Amsterdam, Department of Neurology, Amsterdam Neuroscience, Vrije Universiteit Amsterdam, Amsterdam UMC, Amsterdam, The Netherlands; 3 Department of Radiology and Nuclear Medicine, Vrije Universiteit Amsterdam, Amsterdam UMC, Amsterdam, The Netherlands; 4 Department of Medical Psychology, Amsterdam Public Health Research Institute, University of Amsterdam, Amsterdam UMC, Amsterdam, The Netherlands; 5 GGZ inGeest Specialized Mental Health Care, Amsterdam, The Netherlands; 6 Department of Psychiatry, UMC Utrecht Brain Center, University of Utrecht, Utrecht, The Netherlands; 7 Department of Psychiatry, Amsterdam UMC Location Vrije Universiteit Amsterdam, Boelelaan 1117, Amsterdam, The Netherlands; 8 Amsterdam Neuroscience, Mood, Anxiety, Psychosis, Sleep & Stress Program, Amsterdam, The Netherlands; 9 Clinical Memory Research Unit, Lund University, Lund, Sweden; 10 EQT Life Sciences Partners, Amsterdam, The Netherlands

**Keywords:** FTD, Psychiatry, Diagnostics, Biomarkers, FDG PET

## Abstract

**Background:**

Behavioural symptoms and frontotemporal hypometabolism overlap between behavioural variant of frontotemporal dementia (bvFTD) and primary psychiatric disorders (PPD), hampering diagnostic distinction. Voxel-wise comparisons of brain metabolism might identify specific frontotemporal-(hypo)metabolic regions between bvFTD and PPD. We investigated brain metabolism in bvFTD and PPD and its relationship with behavioural symptoms, social cognition, severity of depressive symptoms and cognitive functioning.

**Results:**

Compared to controls, bvFTD showed decreased metabolism in the dorsal anterior cingulate cortex (dACC) (*p *< 0.001), orbitofrontal cortex (OFC), temporal pole, dorsolateral prefrontal cortex (dlPFC) and caudate, whereas PPD showed no hypometabolism. Compared to PPD, bvFTD showed decreased metabolism in the dACC (*p *< 0.001, *p *< 0.05_FWE_), insula, Broca’s area, caudate, thalamus, OFC and temporal cortex (*p *< 0.001), whereas PPD showed decreased metabolism in the motor cortex (*p *< 0.001). Across bvFTD and PPD, decreased metabolism in the temporal cortex (*p *< 0.001, *p *< 0.05_FWE_), dACC and frontal cortex was associated with worse social cognition. Decreased metabolism in the dlPFC was associated with compulsiveness (*p *< 0.001). Across bvFTD, PPD and controls, decreased metabolism in the PFC and motor cortex was associated with executive dysfunctioning (*p *< 0.001).

**Conclusions:**

Our findings indicate subtle but distinct metabolic patterns in bvFTD and PPD, most strongly in the dACC. The degree of frontotemporal and cingulate hypometabolism was related to impaired social cognition, compulsiveness and executive dysfunctioning. Our findings suggest that the dACC might be an important region to differentiate between bvFTD and PPD but needs further validation.

**Supplementary Information:**

The online version contains supplementary material available at 10.1186/s13550-023-01020-2.

## Introduction

Frontotemporal dementia (FTD) is the second most common cause of young onset dementia [1] and has several clinical phenotypes, including the behavioural variant of FTD (bvFTD), which is the most prevalent. BvFTD is marked by early impairment in social cognition and behavioural symptoms such as disinhibition, compulsions, apathy and a changed eating pattern [2].

In the absence of an accurate specific molecular biomarker for bvFTD, the disease is particularly difficult to distinguish from primary psychiatric disorders (PPD), such as late-onset bipolar disorder, autism spectrum disorder, personality disorders, schizophrenia and major depressive disorders, due to significant overlap in clinical symptoms such as apathy, disinhibition and stereotyped/compulsive behaviour [3]. Distinction is further hampered by relatively subtle or mild atrophy observed on structural imaging in early disease stages of bvFTD (50%) and age-related frontal lobe atrophy [4–6].

Due to this diagnostic challenge, approximately 50% of bvFTD patients are misdiagnosed with a psychiatric diagnosis, inducing a delay of approximately 5–6 years before a correct diagnosis is established [7, 8]. Accurate diagnosis is important to adequately inform caregivers regarding disease course and prognosis and is essential for correct patient selection in future clinical trials. While bvFTD patients are now over-treated with psychotropic drugs that can cause adverse side effects negatively influencing quality of life [9], psychiatric patients whom are misdiagnosed as bvFTD are also not receiving optimal treatment.

Whereas [^18^F]-2-deoxy-2-fluoro-D-glucose (FDG) positron emission tomography ([^18^F]FDG PET) has a high sensitivity for diagnosis of bvFTD, patients with PPD with late-onset behavioural symptoms that closely resemble bvFTD can as well show frontotemporal hypometabolism. In a previous study, we found that 33% of PPD with late-onset behavioural symptoms had a false-positive [^18^F]FDG PET scan for bvFTD, resulting in a low specificity (68%) [6]. In addition, abnormal frontotemporal brain metabolism has been extensively demonstrated in various PPD compared to controls [10–15]. According to the diagnostic criteria of bvFTD, the presence of behavioural symptoms in conjunction with frontotemporal hypometabolism on functional neuroimaging meets the diagnostic criteria for "probable bvFTD" [2], thus easily leading to misdiagnosis in individuals with a primary psychiatric disorder.

Voxel-wise comparisons of (semi)quantitative FDG PET images are a commonly used (diagnostic) measure to investigate brain metabolism and could be a useful adjunct to conventional visual assessment [16]. There are limited FDG PET studies that have performed voxel-wise comparisons in bvFTD and PPD; moreover, these studies did not include unselected patient samples with late-onset behavioural symptoms that reflect clinical reality. Delvecchio et al. [17] found distinct patterns of abnormally decreased metabolism in bvFTD in the left orbitofrontal cortex, superior temporal gyrus, anterior cingulate cortex and caudate compared to PDD. However, these authors only studied a relatively “homogeneous” group of patients with a bipolar disorder, while the psychiatric differential diagnosis with bvFTD is broader, including schizophrenia, autism spectrum disorder, obsessive compulsive disorder, personality disorders and major depressive disorder.

To our knowledge, there are no studies yet which have investigated and compared specific patterns of aberrant brain metabolism between bvFTD and various PPD with late-onset behavioural symptoms by means of voxel-wise comparisons of FDG PET images. Identification of specific brain metabolic patterns may aid to earlier and more accurate distinction between bvFTD and PPD in clinical practice. Also, it would be worthwhile to investigate whether severity of brain hypometabolism is associated with severity of clinical symptoms and cognitive performance. In this study, we aimed to identify specific brain metabolic patterns between bvFTD, PPD with late-onset behavioural symptoms and a cognitively normal control group. Additionally, we investigated whether severity of brain hypometabolism is associated with behavioural symptoms, social cognition, mood and cognitive performance.

## Methods

### Participants

Patients with bvFTD [2], PPD and cognitively normal controls were included with available MRI, [^18^F]FDG PET scans and clinical data (behaviour and mood surveys, social cognition, cognitive performance). BvFTD and PPD patients were participants of the late-onset frontal lobe (LOF) [18] and/or the ongoing Social Brain Project (SBP) cohorts. Both are multicentre prospective observational studies aimed to identify and distinguish early bvFTD from other diseases that are related to the frontal lobe syndrome, such as primary psychiatric disorders. The SBP and LOF largely overlap in study design as described previously [18], yet the SBP has a greater battery for testing several levels of social cognition. Participants were recruited through the memory clinic of the Alzheimer Center of Amsterdam and the Old Age Psychiatry Department of Geestelijke Gezondheidszorg inGeest (GGZinGeest) Amsterdam in the Netherlands, between April 2011 and June 2013 (LOF) and between 2016 and May 2020 (SBP). Inclusion criteria for both studies included gradually evolved late-onset behavioural symptoms (apathy, disinhibition, compulsiveness, hyperorality/dietary changes, impaired social cognition) between 40 and 70 years of age and mini-mental state examination (MMSE) score > 18. Cognitively normal controls were part of the Alzheimer Dementia Cohort (ADC) [19].

### Diagnostic procedure

All participants underwent a standardized diagnostic procedure including a semi structured interview for psychiatric and cognitive symptoms as part of the psychiatric, neurological and physical examination during clinical assessment by both a neurologist and old age psychiatrist. Further, neuropsychological assessment, blood examination to rule out somatic causes, biomarker assessment in cerebrospinal fluid (CSF), electroencephalography (EEG) and neuroimaging (magnetic resonance imaging (MRI) and a [^18^F]FDG PET scan were performed. Diagnosis was established in a multidisciplinary meeting using consensus criteria for probable/definite bvFTD^4^ and PPD [Diagnostic and Statistical Manual of Mental Disorders (DSM-5)] [20].

All bvFTD patients fulfilled diagnostic criteria for *probable* (*n *= 18*)* or *definite* FTD (total *n *= 10, *Chromosome 9 open reading frame 72 (C9ORF72)-mutation* *n *= 7, *Charged multivesicular body protein 2B (CHMP2B)* *n *= 1, *progranulin gene (GRN)* *n *= 2) [2]. In the probable bvFTD group, 16/18 patients were genetically tested and negative for *C9ORF72, microtubule-associated protein tau (MAPT)* or *GRN mutations.* Psychiatric patients (*n *= 35) were diagnosed according to the DSM-5 [20]. Diagnostic classification at the time of the [^18^F]FDG PET included *n *= 11 major depressive disorder, *n *= 6 bipolar disorder (*n *= 3 euthymic phase, *n *= 1 mania, *n *= 1 hypomania, *n *= 1 depressive state), *n *= 3 other specified personality Disorders (with compulsive, dependent and avoidant traits), *n *= 2 autism spectrum disorder, *n *= 2 schizophrenia spectrum and other psychotic disorders, *n *= 1 functional neurological symptom disorder/conversion disorder, *n *= 1 adjustment disorder, *n *= 4 unspecified mental disorder, *n *= 5 multiple primary psychiatric disorders (*n *= 1 major depressive disorder in remissio*n *+ other specified personality disorder (with obsessive compulsive traits), *n *= 1 major depressive disorder + personality traits (unspecified) + Impulse regulation problems, *n *= 1 life phase problems + unspecified personality disorder, *n *= 1 other specified personality disorder (with avoidant traits) + adjustment disorder, *n *= 1 adjustment disorder + posttraumatic stress disorder both in remission. In 30 PPD patients, a genetic mutation (*C9ORF72, MAPT, GRN*) was excluded. The median (clinical) follow-up duration in years was 4 [2–7] in bvFTD and 3 [2–5] years in PPD, respectively, during which their diagnosis was re-confirmed (Table 1). For accurate patient selection, the most recent clinical diagnosis at follow-up was used. In PPD cases, the diagnosis at the time of the [^18^F]FDG PET scan was used for specifying the state of the psychiatric disorder, for example: the last diagnostic conclusion at follow-up revealed that the patient had a Bipolar Disorder. In hindsight, the patient was in a manic state during the [^18^F]FDG PET scan. Thus, in case of confirmed PPD diagnosis after 3 years during clinical follow-up, we used the psychiatric diagnosis and state at the time of admission of the [^18^F]FDG PET scan to enable the most reliable interpretation of metabolic patterns.

**Table 1:** Demographics

	All	bvFTD*	PPD^¥^	Controls	*p* value
	*n *= 79	*n *= 28	*n *= 35	*n *= 16	
Sex (Female, %)	*25 *(31)	11 (38)	8 (23)	6 (38)	0.34^†^
Age (median, Q1–Q3)	62 [57–68]	63 [57–69]	60 [55–64]	68 [60–71]	0.01^∞a,b^
Education^1^	4.9 ± 1.3	5.2 ± 1.4	4.6 ± 1.2	5.8 ± 1.3	0.01†^a,b^
Disease duration (median, Q1–Q3)^2^	3.0 [2–5]	2.5 [2–6]	3.0 [2–5]	n.a	0.71^$^
Follow-up duration, years (median, Q1–Q3)	3 [2–5]	4.0 [2–7]	3 [2–5]	n.a.^@^	0.32^$^
Psychotropic drug use (*n* yes, %)	35 (44)	16 (55)	19 (54)	0 (0)	0.80^†^^
Combination of drugs*	15 (19.0)	4 (14.3)	11 (31.4)	0 (0)	
SSRI/SNRI	10 (12.7)	5 (17.9)	5 (14.3)	0 (0)	
TCA	2 (2.5)	0 (0)	2 (5.7)	0 (0)	
Atypical anti-psychotics	2 (2.5)	1 (3.6)	1 (2.9)	0 (0)	
Benzodiazepines	1 (1.3)	1 (3.6)	0 (0)	0 (0)	
Other**	5 (6.3)	3 (10.7)	2 (5.7)	0 (0)	
MMSE (median, Q1–Q3)	27 [26–29]	26 [24–29]	27 [26–29]	29 [28–30]	<0.001^∞a,b^
FAB (median, Q1–Q3)	17 [14–18]	16 [14–17]	17 [12–18]	18 [17, 18]	0.02^∞a,b^
Ekman 60 faces test (mean ± SD)	37 ± 9.0	32 ± 9.3	40 ± 7.1	^^	0.004^#c^
MADRS (mean ± SD)	12 ± 8.9	7 ± 6.5	15 ± 9.0	^^	0.006^#c^
SRI (median, Q1–Q3)	5 [2–13]	11 [3–17]	4 [2–8]	^^	0.045∞^c^
*Executive functioning/language*					
Letterfluency D-A-T (median, Q1–Q3)	28 [18–35]	21 [16–37]	32 [18–36]	^^	0.26^$^
Animal fluency (median, Q1–Q3)	19 [13–24]	14 [9–18]	20 [14–25]	23 [20–30]	0.002∞^a,c^
Trail making test B (median, Q1–Q3)	104 [75–174]	116 [84–239]	99 [74–166]	74 [59–96]	0.009∞^a,b^
Colour-word interference (median, Q1–Q3)	1.8 [1.6–2.0]	1.9 [1.6–2.1]	1.8 [1.6–2.0]	1.8 [1.7–1.9]	0.84∞
Stroop 3 (median, Q1–Q3)	130 [107–156]	140 [117–160]	129 [107–186]	109 [80–140]	0.06∞
[^18^F]FDG PET scans visually assessed, % abnormal	53%	89%	49%	0%	
*FDG SUV normalization variables*					
Body weight, kg (median, Q1–Q3)	78 [69–85]	73 [65–79]	82 [70–96]	80 [67–85]	0.06^∞^
Injected dose, MBq (median, Q1–Q3)	188 [181–195]	190 [181–195]	186 [181–195]	188 [182–191]	0.67^∞^
Mean activity in dACC (mean ± SD)^±^	5.79 ± 1.51	5.01 ± 1.43	6.39 ± 1.48	5.79 ± 1.10	0.003^c^
Mean activity in motor cortex (mean ± SD)^±^	5.26 ± 1.06	5.30 ± 1.12	5.46 ± 1.07	4.71 ± 0.75	0.04^a,b^

Data are representative for time of conducting [^18^F]FDG PET scan

*n.a.* Not applicable, *dACC* dorsal anterior cingulate cortex, *FAB* frontal assessment battery, *MADRS* Montgomery Åsberg Depression Rating Scale, *MBq*megabecquerel, *MMSE* mini-mental state examination, *SSRI* Selective serotonin reuptake inhibitor, *SUV* standardized uptake value, *SNRI* non-selective serotonin reuptake inhibitor/serotonin–norepinephrine reuptake inhibitors, *SRI* Stereotypy Rating Inventory, *TCA* tricyclic antidepressants

*Probable bvFTD *n *= 18, definite bvFTD *n *= 10

*Includes anti-epileptics, classic/atypical anti-psychotics, benzodiazepines, lithium, MAO-inhibitor, TCA

**Includes amphetamines, levodopa with decarboxylase inhibitors, drugs for nicotine addiction, melatonin

^†^Fisher exact

^∞^Kruskal–Wallis test

^$^Mann–Whitney *U* Test

^#^Independent *T*-test

^^^Comparison between bvFTD and PPD

^±^Raw data of ROI, without using proportional scaling

^@^Only seen once at baseline visit

^^^^Not administered in controls

^1^The level of education was classified using the Verhage system [2], ranging from 1 (no or little education) to 7 (highest academic degree)

^2^Represents the time from onset of symptoms till performance of the [^18^F]FDG PET scan

^a^Significant difference between bvFTD versus controls

^b^Significant difference between PPD and controls

^c^Significant difference between bvFTD versus PPD

Additionally, and as a reference group, cognitively normal controls (*n *= 16) were included and had no current or recent psychiatric nor a neurodegenerative disorder based on previously described standardized diagnostic assessments. Excluded were participants with current or recent alcohol abuse prior to the [^18^F]FDG PET scan, repetitive head injury, co-pathology of other forms of dementia, (somatic) comorbidity accounting for behavioural symptoms, or if the MRI/[^18^F]FDG PET scan was of low quality, had motion artefacts > 5 mm, or was performed elsewhere.

### Behavioural symptoms, social cognition, mood and cognitive functioning

The frontal assessment battery (FAB) and mini-mental state examination (MMSE) were used to examine frontal dysexecutive functions and global cognition, respectively. The Stereotypy Rating Inventory (SRI) was used to investigate compulsive and stereotypical behaviour, with higher scores reflecting more and severe compulsive or stereotypical behaviour. Severity of depressive symptoms were assessed using the Montgomery Åsberg Depression Rating Scale (MADRS), with higher scores reflecting more severe depressive symptoms. The Ekman 60 faces test was used to investigate facial emotion recognition, with higher scores reflecting better performance. The following neuropsychological tests were used to assess executive functioning: letter fluency (D-A-T), trail making test B (TMTB, mental flexibility), Stroop 3, colour-word interference (i.e. Stroop 3 corrected for Stroop 2) and language (letter fluency D-A-T, animal Fluency). Higher scores on language tests reflect better executive performance, whereas higher scores on the Trail Making Test B and the Stroop 3 indicate worse executive performance.

All tests were assessed within an interval of less than 12 months between the [^18^F]FDG PET scan and neuropsychological assessment (median 1.7 months). The [^18^F]FDG PET scan was only performed in case of remaining diagnostic uncertainty, and not part of regular study visits of the LOF and SBP study. Therefore some data on behaviour, social cognition, mood and cognitive performance were missing (as indicated, Table 3).

### Positron emission tomography

Fifteen minutes prior to injection of 187.0 ± 8.7 megabecquerel (MBq), subjects were asked to rest in a dimly lit room with minimal background noise and were instructed to keep their eyes closed or wear an eye mask. Next, [^18^F]FDG PET scans were acquired 45 min post-injection for a duration of 15 min (3 frames of each 5 min), head fixation bands were used to reduce micromovement. [^18^F]FDG PET scans were obtained using a Gemini TF 64 PET-CT (Philips Medical Systems, Cleveland, OH, USA) (bvFTD *n *= 14, PPD *n *= 19), ECAT Exact HR + scanner (Siemens/CTI, Knoxville, TN) (bvFTD *n *= 7, PPD *n *= 12, cognitively normal controls *n *= 16) or Ingenuity TF PET/CT (Philips Healthcare, Cleveland, OH, USA) (bvFTD *n *= 7, PPD *n *= 4) scanner. PET data were normalized and corrected for random events, dead time, scatter, and decay. Attenuation correction was performed using a transmission scan (ECAT HR +) or a low-dose CT for PET-CT acquisitions. The reconstruction protocol has been described elsewhere [21], but included standard reconstruction algorithms for both systems (ECAT HR + : standard filtered back-projection; PET-CT 3D row-action maximum likelihood algorithm).

### Magnetic resonance imaging

Brain MR images were obtained on a 3T whole-body MR system (Signa HDxt; GE Medical Systems, Milwaukee, WI, USA) using an 8-channel head coil equipped with foam padding to limit head motion. A standardized MRI acquisition protocol for memory clinic patients was used [19] and included a sagittal 3D heavily T1-weighted gradient-echo sequence with coronal reformats, a sagittal 3D T2-weighted fluid-attenuated inversion-recovery (FLAIR) fast spin-echo with axial reformats, a transverse T2-weighted fast spin-echo, a transverse T2* susceptibility sequence, and diffusion-weighted imaging/EPI. Sequences were performed using 3 mm slices/reformats with 1 mm in-plane resolution and provided whole-brain coverage. MRI and [^18^F]FDG PET scan interval was restricted to a maximum of 12 months for controls (median interval: 0 months) and PPD (median interval: 1 month), and 9 months for FTD (median interval: 1 month) to ensure optimal co-registration and quantification.

### Image preprocessing and analysis

[^18^F]FDG PET images were adjusted for body weight and injected activity to obtain standardized uptake value (SUV_BW_) images using an in-house engineered software tool for kinetic and parametric analyses of dynamic PET studies (PPET) [22]. Image preprocessing and between-group analysis were performed with SPM12 in MATLAB (MathWorks, Release 2017b). SUV_BW_ images were co-registered to the MR T1 image and then warped to the Montreal Neurological Institute space after which images were smoothed using an 8-mm full-width-at-half maximum Gaussian kernel in SPM12 (Statistical Parametric Mapping; Wellcome Trust Centre for Neuroimaging, London, UK). As bvFTD and PPD show overlapping frontotemporal hypometabolic patterns [8] we used a pre-defined “FTD mask” including all (grey matter) frontal, temporal, anterior cingulate cortical regions and the thalamus (with dilatation of 1 mm), generated in a single "mask image" with an automated anatomical labelling (AAL) brain atlas in WFU PickAtlas, for further statistical analysis in SPM. Brain regions were based upon functional neuroimaging studies in bvFTD and PPD [10–15, 23–28].

In order to perform group analyses and to allow comparisons in all brain regions, SUV_BW_ images were normalized to global uptake using proportional scaling in SPM12 [29]. Voxel-wise comparisons were carried out while using a grey matter mask and mean global calculation in SPM12. Also, as a methodological check, we performed two validations to re-assure that our finding were not driven by proportional scaling: 1) analysis were repeated without using proportional scaling and 2) we created regions of interest (ROI) of our most important brain regions, in which the mean SUV_BW_ activity (without scaling) was extracted of each subject and subsequently the activity between groups was compared (data in Additional file 1: Fig. S1).

### Statistical analyses

Statistical analyses of demographic and clinical data were performed in IBM SPSS Statistics for Windows version 26.0. Depending on the distribution, demographic and clinical data were analysed with analysis of variance (ANOVA) or Kruskal–Wallis test. In case of categorical variables, Fisher’s exact test was applied.

We firstly performed voxel-wise group comparisons on FDG SUV_BW_ in an a priori defined FTD mask between bvFTD, PPD and cognitively normal controls while adjusting for age, sex, scanner, psychotropic drug use (yes/no) in SPM12. Furthermore, to ensure that genetic FTD cases have not driven our findings, we additionally performed a sensitivity analyses by repeating our analyses while excluding genetic FTD cases (*n *= 18).

We secondly investigated associations between brain metabolism and behavioural symptoms, depressive symptoms, social cognition and cognitive performance in separate statistical models (for each test), and adjusted for age, sex and scanner. In case of cognitive testing we also adjusted for education level based on the Dutch Verhage system [30] (range: 1 no or little education to 7 (academic degree or higher). Regression analyses were performed within the a priori defined FTD mask, across all groups. Diagnostic groups were combined to increase statistical power and to assess whether FDG PET can provide transdiagnostic value for late-onset symptoms, i.e. independent for diagnosis. Additionally, exploratory regression analysis per diagnostic group was performed (data in Additional file 1). Results were considered significant if the voxels survived a threshold of *p *< 0.001 (cluster size *k* ≥ 10), and/or if exceeded a more conservative threshold set at *p *< 0.05 corrected for multiple comparisons using family-wise error (FWE). In addition, an exploratory whole-brain analysis (with conservative threshold, i.e. FWE) was performed to investigate metabolism beyond the FTD mask (Additional file 1: Fig. S2, Table 1) to reduce the risk of false negatives and because hypometabolism may occur in other brain regions [31–34]. For visual purposes, we generated *T*-maps displayed in BrainNet Viewer [35]. Furthermore, effect sizes per brain region were calculated using the Cohen’s *d* across all group comparisons to provide a more robust statistical measure of the magnitude of differences observed between groups. The Cohen’s *d* was calculated based upon the sample size and SPM-derived T values, while using the following formula: Cohen’s *d* = *t*/[*N*]^1/2^.

## Results

### Demographics

There were no age differences between PPD and bvFTD, but both were younger (*p *= 0.01) than controls. There were no differences with respect to disease duration between bvFTD and PPD (*p *= 0.71). Across bvFTD, PPD and controls, there were no differences in distribution of sex (*p *= 0.34), weight (kg) (*p *= 0.06) or mean injected dose (MBq) (*p *= 0.67). Compared to PPD, bvFTD patients performed worse on facial emotion recognition (Ekman 60 faces test mean ± SD: bvFTD 32 ± 9.3 vs. PPD 40 ± 7.1, *p *= 0.004) and had more severe compulsive symptoms (SRI median, Q1–Q3: bvFTD 11 [3–17] vs. PPD 4 [2–8], *p *= 0.045). PPD experienced more severe depressive symptoms compared to bvFTD (MADRS mean ± SD: PPD 15 ± 9.0 vs. bvFTD 7 ± 6.5, *p *= 0.006). None of the controls used psychotropic drugs at the time of study (Table 1).

### *Brain metabolism in *a priori* defined FTD mask*

**Fig. 1:**
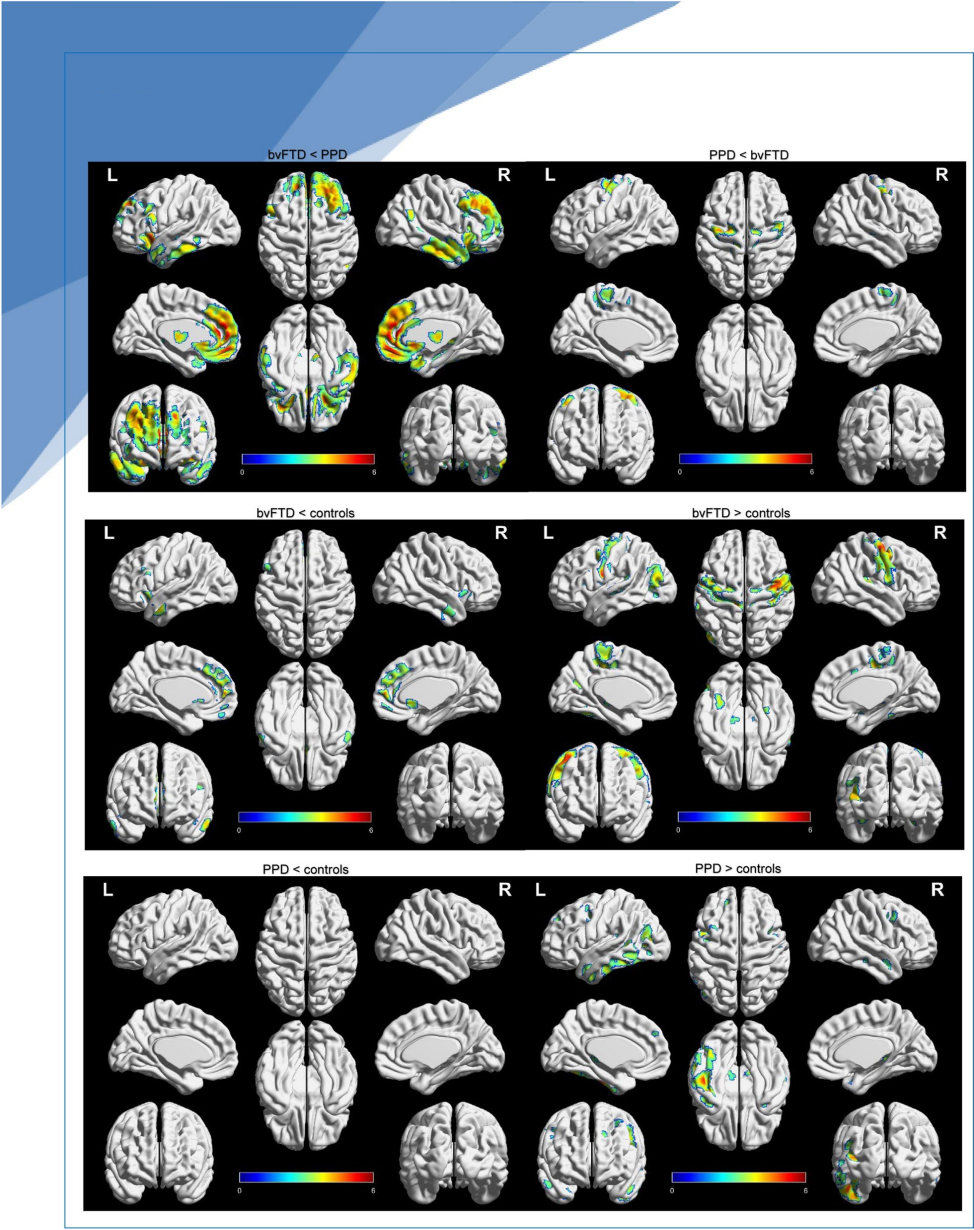
Functional brain abnormalities of FTD and PPD compared to controls. Abbreviations: bvFTD = behavioural variant of frontotemporal dementia; PPD = primary psychiatric disorders. Patterns of brain metabolism of FTD and PPD patients versus cognitively normal controls, adjusted for age, sex, psychotropic drugs, scanner type. Surface rendering of significant voxels from contrasts between FTD, PPD patients and controls are displayed at *p *< 0.005 for visual purposes, extent threshold *k *= 0. Analysis of bvFTD versus controls was not adjusted for psychotropic drug use due to a small sample size. Significant clusters are displayed in Table 2. Images were created with BrainNet [35]

We first investigated brain metabolism across groups (Fig. 1; Table 2). Compared to controls, bvFTD showed decreased metabolism in the bilateral dorsal anterior cingulate cortex (dACC), the left orbitofrontal cortex (OFC) and left temporal pole, and right dorsolateral prefrontal cortex (dlPFC) and right caudate (all *p *< 0.001). In contrast, bvFTD showed increased metabolism in the bilateral motor cortex and left superior temporal gyrus compared to controls (all *p *< 0.001).

**Table 2:** Voxel-wise contrasts of brain metabolism within the FTD mask in bvFTD, PPD and controls

Anatomical region	Laterality	MNI coordinates			Cohen’s *d*	Cluster size in voxels
		*x*	*y*	*z*		
*bvFTD* < *PPD*						
Dorsal ACC	Bilateral	− 2	46	16	0.75	3953
Insula	Left	− 38	20	− 8	0.61	625
Caudate	Right	6	18	− 2	0.60	120
Broca area	Left	− 50	16	26	0.49	81
Thalamus	Right	4	− 20	8	0.53	232
Temporal cortex						
Inferior temporal gyrus	Right	58	− 18	− 26	0.53	560
Fusiform gyrus	Left	− 58	− 40	− 16	0.44	87
Temporal pole	Bilateral	− 46	10	− 30*	0.49	20
*bvFTD* > *PPD*						
Motor cortex (pre-motor/SMA)	Left	− 36	− 12	62	0.56	160
	Right	42	− 6	54	0.52	42
*bvFTD* < *controls*^*a*^						
Dorsal ACC	Bilateral	2	48	6	0.70	99
OFC	Left	− 42	18	− 8	0.53	33
dlPFC	Right	0	30	36	0.55	35
Temporal pole	Left	− 62	3	− 20	0.60	36
Caudate	Right	6	18	− 2	0.57	10
*bvFTD* > *controls*^*a*^						
Thalamus	Left	18	− 22	6	0.77	63
Motor cortex						
Pre-motor/SMA	Right	40	− 10	58	0.76	391
Pre-motor/SMA	Left	− 56	− 2	20	0.74	236
Primary motor cortex	Right	38	− 30	64	0.67	21
Primary auditory cortex	Right	44	− 28	8	0.56	73
*PPD* > *controls*						
Temporal cortex						
Inferior temporal gyrus	Left	− 46	− 24	− 28	0.69	106
Fusiform gyrus	Left	− 56	− 42	− 16	0.53	25
Temporal pole	Left	− 40	4	− 40	0.50	17
Thalamus	Right	18	− 29	4	0.51	22

Significance set at *P *< .001, cluster size *k* > 10, FWE-uncorrected. Results are adjusted for age, sex, psychotropic drug use and type of scanner. The Cohen’s *d* was calculated based upon the sample size and SPM-derived *T* values, while using the following formula: Cohen’s* d* = *t*/[*N*]^1/2^. Interpretation of the effect size of Cohen’s *d* are as follows: 0.2 = small effect. 0.5 = moderate effect. 0.8 = large effect

*ACC* anterior cingulate cortex, *dlPFC* dorsolateral prefrontal cortex, *OFC* orbitofrontal cortex, *MNI* Montreal Neurological Institute, *PFC* prefrontal cortex, *SMA* Supplementary motor area

*MNI coordinates of left pole are displayed

^a^adjusted for age, sex, type of scanner (not for psychotropic drug use due to smaller sample size)

Compared to controls, PPD showed increased metabolism in the left temporal cortex (temporal pole, inferior and fusiform gyrus), but PPD did not show hypometabolism compared to controls.

Compared to PPD, bvFTD showed decreased metabolism in the bilateral dACC (*p *< 0.001, *p *< 0.05_FWE_) and temporal cortex (right inferior gyrus and pole, left fusiform gyrus), the left insula and left Broca area, the right caudate and right thalamus (all *p *< 0.001). By contrast, PPD showed decreased metabolism in the bilateral motor cortex (all *p *< 0.001) compared to bvFTD. Both bvFTD and PPD showed increased metabolism in the right thalamus compared to controls (all *p *< 0.001) and PPD showed the relative highest levels of thalamic metabolism across groups. If we repeated analysis, while excluding genetic FTD cases, we found largely comparable results (Additional file 1: Table S2). More specifically, in sporadic FTD, we found hypometabolism in the medial temporal gyrus (MTG), dACC and temporal pole (all *p *< 0.001, *p *< 0.05_FWE_) compared to PPD, and MTG hypometabolism compared to controls (all *p *< 0.001, *p *< 0.05_FWE_).

### Brain metabolism in relation to behavioural symptoms, social cognition and cognitive functioning

**Fig. 2:**
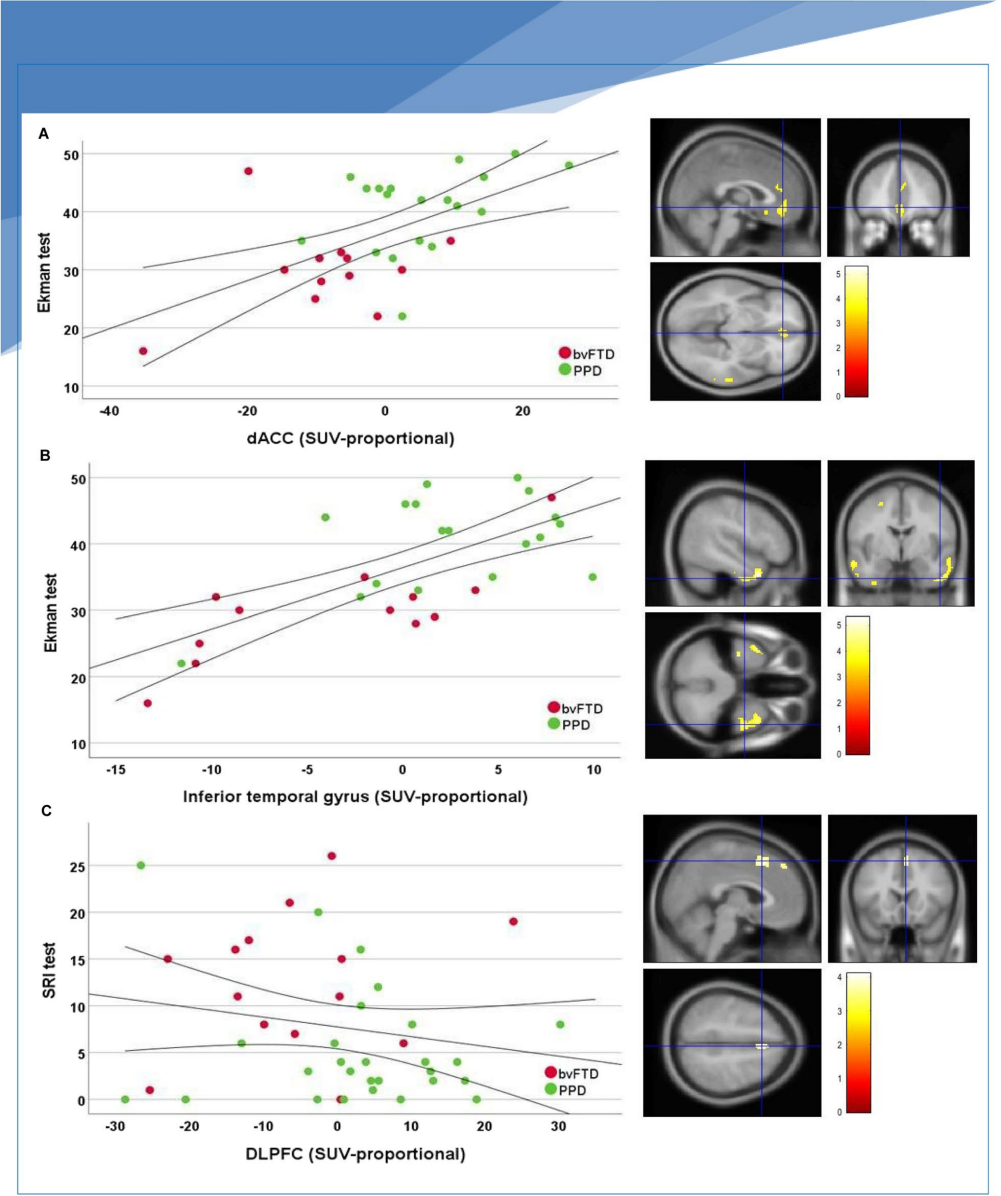
Association of brain metabolism and cognitive tests. Abbreviations: dACC = dorsal anterior cingulate cortex. dlPFC = dorsolateral prefrontal cortex. Ekman test = Ekman 60 faces test. SRI = Stereotypy Rating Inventory. Scatterplots display **A** Association of brain metabolism in the dACC and Ekman 60 faces test (facial emotion recognition). Lower scores on the Ekman 60 faces test denote worse facial emotion recognition **B** Association of brain metabolism in the inferior temporal gyrus and Ekman 60 faces test (facial emotion recognition) **C** Association of brain metabolism in dlPFC and compulsive behaviour. Higher scores on the SRI denote more and severe compulsive behaviour. Curved lines represent mean confidence intervals. Right side of the figure: corresponding visual representation of patterns of metabolism of FTD and PPD patients that associate with facial emotion recognition and compulsive behaviour. All voxels are scaled to the mean uptake using proportional scaling, thus negative values reflect lower mean values relative to the global uptake. Surface rendering of significant voxels from contrasts of FTD and PPD patients are displayed at *p *< 0.001, all significant clusters are displayed in Table 3. Images were created with SPM12

Table 3 and Fig. 2 present the associations between brain metabolism and behavioural symptoms, social cognition and cognitive functioning. Across bvFTD and PPD, decreased metabolism in the bilateral temporal cortices (inferior gyrus; *p *< 0.001, *p *< 0.05_FWE_, medial and fusiform gyrus and left pole; *p *< 0.001), bilateral dACC, right dlPFC, right OFC and right motor cortex (all *p *< 0.001) was associated with worse facial emotion recognition. In addition, decreased brain metabolism in the right dlPFC was associated with more severe compulsive behaviour (all *p *< 0.001) and decreased brain metabolism in the right PFC and left motor cortex was associated with worse letterfluency. Across bvFTD, PPD and controls decreased metabolism in the right PFC and left temporal pole was associated with worse animal fluency (all *p *< 0.001), and decreased metabolism in the left thalamus was associated with more colour-word interference (*p *< 0.001). There were no associations between brain metabolism, mental flexibility (TMTB) or severity of depressive symptoms (MADRS).

**Table 3:** Association brain metabolism within the FTD mask and behavioural symptoms, social cognition and cognitive function

Anatomical region	Laterality	MNI coordinates			Cohen’s *d*	Cluster size in voxels
		*x*	*y*	*z*		
*Social cognition (Ekman 60 faces test)*						
Temporal cortex						
Inferior temporal gyrus	Right	48	− 4	− 38	0.98	941
Temporal pole	Left	− 48	12	− 34	0.88	519
Fusiform gyrus	Left	− 40	− 36	− 24	0.75	26
Medial temporal gyrus	Right	62	− 26	− 4	0.75	89
Dorsal ACC	Bilateral	2	42	− 10	0.77	140
dlPFC	Right	10	− 30	46	0.72	15
OFC	Right	2	42	− 8	0.67	66
Motor cortex	Left	− 26	− 4	56	0.72	15
*Compulsiveness*						
dlPFC	Right	4	18	52	0.64	129
*Executive function and language*						
Letterfluency D-A-T						
PFC^^^	Right	10	68	14	0.57	321
Motor cortex	Left	− 10	26	62	0.50	26
Animal fluency						
PFC	Right	2	46	16	0.54	65
Temporal pole	Left	− 60	4	− 22	0.50	26
Colour-word interference						
Thalamus	Left	− 6	– 18	2	0.44	10

Significance was set at *P *< .001, cluster size *k *> 10, FWE**-**uncorrected. Analysis for social cognition and compulsiveness were adjusted for age, sex and type of scanner. Analysis for executive function and language were adjusted for age, sex, education and type of scanner

*ACC* anterior cingulate cortex, *dlPFC* dorsolateral prefrontal cortex, *OFC* orbitofrontal cortex, *MNI* Montreal Neurological Institute

^^^Anterior part of PFC. The Cohen’s *d* was calculated based upon the sample size and SPM-derived *T* values, while using the following formula: Cohen’s* d* = *t*/[*N*]^1/2^. Interpretation of the effect size of Cohen’s *d* are as follows: 0.2 = small effect. 0.5 = moderate effect. 0.8 = large effect. Overview of patients included per test based on available data: social cognition (Ekman 60 faces test): bvFTD: 11/28; PPD: 18/35. There were no differences regarding demographics, clinical data and cognitive performance between the samples with and without missing data. Compulsiveness (SRI): bvFTD 14/28; PPD 27/35. Executive function and language: Letterfluency D-A-T: bvFTD 25/28; PPD 26/35. Animal fluency: bvFTD 25/28; PPD 27/35; controls: 8/16. Colour-word interference: bvFTD 24/28; PPD 26/35; controls 7/16. Yielded no correlations with brain metabolism, thus not shown in Table 3: mental flexibility (TMTB): bvFTD 25/28; PPD 27/35; controls 8/16. Severity of depressive symptoms (MADRS): bvFTD 14/28; PPD: 27/35

While the results need to be interpreted with caution due to the sample size, separate correlation analysis per diagnostic group are reported in the Additional file 1, section “Brain metabolism associated with behavioural symptoms, social cognition and cognitive functioning—per diagnostic group”.

### Exploratory analysis

To explore whole-brain metabolism beyond the FTD mask, we repeated analyses (all results in the Additional file 1: Fig. S2, Table S1). Compared to controls, bvFTD showed increased metabolism in the bilateral brain stem (*p *< 0.05_FWE_). Compared to controls and PPD, bvFTD showed increased metabolism in the bilateral cerebellum (*p *< 0.05_FWE_).

## Discussion

Our findings provide evidence for subtle but distinct brain metabolic patterns between bvFTD, PPD and controls with the dACC as key hypometabolic region in bvFTD. Moreover, the degree of hypometabolism in the dACC and frontotemporal regions was related to impaired social cognition, compulsive behaviour and executive dysfunctioning providing transdiagnostic evidence for hypometabolism to play a role in frontal dysexecutive symptoms.

This is the first voxel-wise [^18^F]FDG PET study to investigate bvFTD in comparison with various PPD with late-onset behavioural symptoms that resemble bvFTD. Complex differential diagnosis is illustrated by Kerssens et al., [36] that described a case of a 54-year-old woman fulfilling criteria for both late-onset schizophrenia and probable bvFTD with bilateral frontotemporal hypometabolism on [^18^F]-FDG PET but with a post-mortem diagnosis of schizophrenia.

Our main finding is that the dACC appears to be a key hypometabolic region in bvFTD compared to both PPD and controls. In a second set of analyses, we additionally found that the degree of dACC hypometabolism is associated with impaired social cognition, which is one of the most distinctive clinical symptom in bvFTD. Previous MRI and PET studies found reduced and grey matter volumes and metabolism in the dACC in bvFTD patients, but we now extend this with FDG PET in a heterogeneous patient sample including bvFTD and various psychiatric disorders. In the present study, we provide evidence for the involvement of the dACC metabolism in bvFTD disease pathology [23–26, 37–39]. The dACC is one of the earliest affected brain regions in bvFTD and is crucially involved in social cognition, executive functioning and decision-making, and dACC functioning has been associated with impaired social cognition [23, 40, 41]. Interestingly, the dACC contains the highest density of von Economo neurons (VENs), a type of large projection neurons that are considered to play a crucial role in social cognition and emotional processing, and that the loss of VENs is associated with the onset of bvFTD [42, 43]. In bvFTD, reduced dACC metabolism may reflect FTD pathology including a loss of VENs.

Besides hypometabolism in the dACC, we found decreased metabolism in the caudate, OFC, dlPFC and temporal pole in bvFTD. In the separate, sensitivity analysis, including only sporadic FTD cases results were replicated. Moreover, we found group differences between FTD and PPD became somewhat more extensive while excluding genetic FTD patients, and showing additional involvement of the medial temporal gyrus. Our findings are in line with literature [17, 29, 31, 44], showing that bvFTD pathology is covering the entire frontotemporal cortex and we extend this in a sample of patients with late-onset behavioural symptoms.

While in bvFTD aberrant brain metabolism in frontotemporal regions seems consistent and diffuse in the present study, in PPD this tends to be more subtle or absent and confined to the bilateral orbitofrontal regions which was exclusively observed in the whole-brain analysis, rather than in the pre-determined FTD mask. The discrepancy between quantitative voxel-wise comparisons and qualitative approaches visually assessed scans is notable. To rule out that differences could be explained by certain quantification techniques, we performed several quantification procedures (with and without scaling), which have generated consistent findings (Additional file 1: Fig. S1). Considering the difference between the qualitative and quantitative results of the PPD versus control group, it is conceivable that heterogeneity and a relatively lower neurobiological factor (including both hypo- and hypermetabolism) in PPD (psycho) pathology contributes to the discrepancy between visual and quantitative assessments. In addition, lumping various PPD psychopathology with different hypometabolic patterns at group level, frontotemporal hypometabolism in PPD may appear less consistent on a voxel level. Furthermore, PPD patients received psychotropic drugs while undergoing the FDG PET scan, and despite applying statistical corrections, this may have influenced our results. Future studies should further investigate disease specific metabolic patterns for PPD versus FTD with late-onset behavioural symptoms including larger sample sizes enabling stratified analysis for psychiatric subgroups.

The majority (49%) of the psychiatric patients in the present study were diagnosed with mood disorders, including bipolar disorder. Our study is in line with Delvecchio et al. [17] that already showed dACC hypometabolism in bvFTD compared to bipolar disorder and cognitively normal controls. Future studies should further investigate whether the dACC represents a suitable region to assess on [^18^F]FDG PET scans in clinical practice when differentiating between bvFTD and primary psychiatric disorders.

Clinically, we found that bvFTD patients performed worse on facial emotional recognition compared to PPD. In a second set of analyses, we investigated whether brain metabolism was associated with social cognition and we found that lower orbitofrontal and temporal (superior, medial and fusiform gyrus) metabolism was associated with worse facial emotional recognition. While it needs to be interpreted with caution due to a relatively sample size, it seems that FTD patients were particularly driving these results. In bvFTD, early impairment of social cognition is a key feature of the disease [45], while in psychiatric disorders there are also "state-dependent" difficulties in attribution of emotion to facial expressions [46–50], that are linked to abnormal neural activation the fusiform, inferior temporal gyrus and orbitofrontal cortex [48, 50]. Our findings are in line with studies that show involvement of orbitofrontal brain regions and fusiform gyrus in recognition of facial, auditory and emotional stimuli [45, 51–53]. Facial emotion recognition relies on various brain regions involved in visual analysis of facial features, analysis of facial identity and expression and emotion decoding, providing a potential explanation for our finding on the fusiform gyrus [54]. In line with our findings, previous studies found that temporal gyrus and OFC atrophy to be associated with face processing and emotion recognition in neurodegenerative disorders such as bvFTD and semantic dementia [45, 53, 55, 56].

Interestingly, additional exploratory whole brain analysis, showed increased metabolism in the brainstem in bvFTD. While its implication needs further investigation in future studies, these findings are in line with previous studies [32, 33, 57, 58] showing brainstem hypermetabolism in FTD and amyotrophic lateral sclerosis (ALS) patients. Studies have demonstrated that proliferating astrocytes and microglia (gliosis), which are associated with neuronal degeneration in FTD, might contribute to the hypermetabolic signal by consuming glucose [57, 59, 60]. Another pathophysiological explanation for our findings could be that hypermetabolic patterns in bvFTD are a compensatory mechanism of brain dysfunction in these brain regions, precipitating hypometabolism and neuronal loss, which has previously been suggested as hypothesis in other neurodegenerative diseases including Huntington [61] and Parkinson’s disease [62]. In PPD, we found increased metabolism in temporal regions and the thalamus, aligning with previous studies that found hypermetabolic patterns in cortico-striato-thalamic feedback loops in PPD [11, 12, 14]. Interestingly, the involvement of over-activated glia is also reported in the pathophysiology of psychiatric disorders [63].

Strengths of the present study are our voxel-wise comparisons of parametric images that enabled detailed and sensitive identification of specific metabolic patterns, beyond visual assessment. Also, in this sample, many of the MRIs were inconclusive with regard to diagnosis, and hence FDG may offer important information in addition to structural imaging. Nonetheless, we acknowledge the potential risk of circularity in our patient selection, as only patients that received a [^18^F]FGD-PET scan were selected, because of remaining diagnostic uncertainty due to an inconclusive MRI. However, we believe that these patients in particular are at risk of misdiagnosis due to overlapping metabolic frontotemporal abnormalities on FDG PET between FTD and PPD and thereby reflecting clinical practice. For these reasons, further validation is necessary in a larger, prospective cohort, including also relatively "clear-cut" FTD and PPD cases, focusing on abnormal metabolic patterns and assessing their diagnostic value by means of sensitivity and specificity, in conjunction with other biomarkers. A limitation is that we included a heterogeneous sample of PPD. Notwithstanding, all patients were included based on comparable “phenotype” with late-onset behavioural symptoms, hence reflecting a realistic clinical scenario. This study incorporated a relatively small sample size from a tertiary referral centre, which could potentially limit the generalizability of the results. Notwithstanding, despite a relatively small and heterogeneous sample, our findings provided consistent evidence for involvement of specific brain areas, such as the dACC. In addition, all patients had a clinical median follow-up of 3 years, which lowers the risk of misdiagnosis, and increased the validity of the present comparisons including complex differential diagnosis. Further, current methodologies employed in this study may not directly be translated into routine clinical practice, but will give novel directions for visual assessment of FDG PET scans in patients with late-onset behavioural symptoms. Future multicentre studies using autopsy confirmed cohorts are needed to establish reproducible procedures and clinical cut-offs of brain hypometabolism, facilitating the discrimination between bvFTD and PPD within clinical contexts.

Finally, we used different scanner types across bvFTD and PPD while controls were all scanned on ECAT Exact HR + scanner (Siemens/CTI, Knoxville, TN), potentially influencing brain metabolism, but we did covary our analyses for scanner type.

## Conclusions

In conclusion, our findings of distinct regions of altered brain metabolism in bvFTD and PPD relate to symptom severity and show that the dorsal ACC in particular might be a suitable region of interest to provide diagnostic distinction of bvFTD and PPD in clinical practice and should be further assessed in a prospective setting.

## Supplementary Information

**Additional file 1.** Region of interest SUV activity across groups.

## Data Availability

The data sets generated during and/or analysed during the current study are available from the corresponding author on reasonable request.

## Abbreviations

AAL: Automated anatomical labelling
ADC: Alzheimer dementia cohort
ANOVA: Analysis of variance
bvFTD: Behavioural variant of frontotemporal dementia
CHMP2B: Charged multivesicular body protein 2B
C9ORF72: Chromosome 9 open reading frame 72
CSF: Cerebrospinal fluid
dACC: Dorsal anterior cingulate cortex
dlPFC: Dorsal lateral prefrontal cortex
DSM: Diagnostic and statistical manual of mental disorders
EEG: Electroencephalography
FAB: Frontal assessment battery
[^18^F]FDG PET: [^18^F]-2-deoxy-2-fluoro-d-glucose (FDG) positron emission tomography
FLAIR: Fluid-attenuated inversion-recovery
FWE: Family-wise error
GGZinGeest: Geestelijke gezondheidszorg ingest
GRN: Progranulin gene
LOF: Late-onset frontal lobe
MADRS: Montgomery Åsberg depression rating scale
MAPT: Microtubule-associated protein tau
MBq: Megabecquerel
MMSE: Mini-mental state examination
MNI: Montreal neurological institute
MRI: Magnetic resonance imaging
OFC: Orbitofrontal cortex
PPD: Primary psychiatric diseases
PPET: Parametric analyses of dynamic PET studies
ROI: Region of interest
SBP: Social Brain Project
SMA: Supplementary motor area
SNRI: Non-selective serotonin reuptake inhibitor/serotonin–norepinephrine reuptake inhibitors
SPM: Statistical parametric mapping
SRI: Stereotypy rating inventory
SSRI: Selective serotonin reuptake inhibitor
SUV: Standardized uptake value
TCA: Tricyclic antidepressants
TDP-43: TAR DNA-binding protein 43
TMTB: Trail making test B
UMC: Universitair Medisch Centrum
VENs: von Economo neurons
