# Supplementary material for: Altered brain metabolism in frontotemporal dementia and psychiatric disorders: involvement of the anterior cingulate cortex
Source: EJNMMI Res. 2023 Jul 26;13:71. doi: 10.1186/s13550-023-01020-2 (PMC10371967; doi:10.1186/s13550-023-01020-2)
Supplement: Supplementary file 1 — Additional file 1. Region of interest SUV activity across groups. [file 13550_2023_1020_MOESM1_ESM.docx]

**Supplementary materials**

**Methods validation**To ensure our findings were not driven by proportional scaling, analysis were repeated without using proportional scaling, while extracting unscaled mean SUV_BW_ activity in our a priori defined regions of interest, and subsequently, activity was compared between groups (Supplementary materials, Figure 1), which showed comparable results.


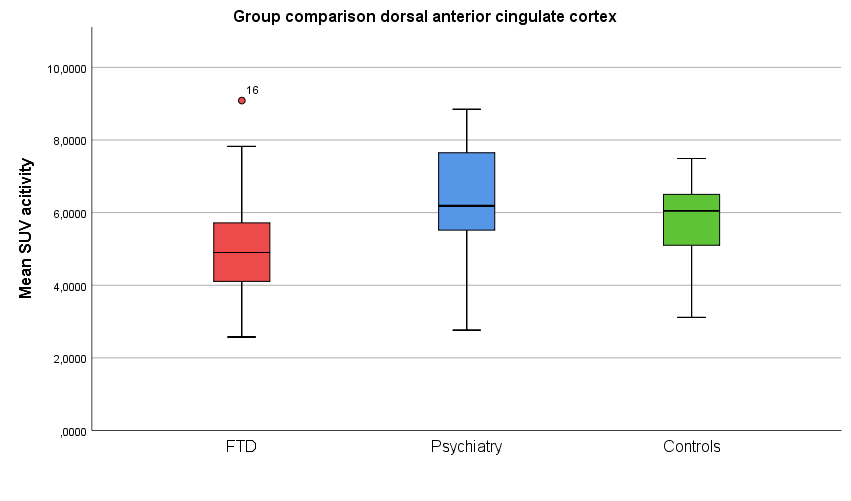

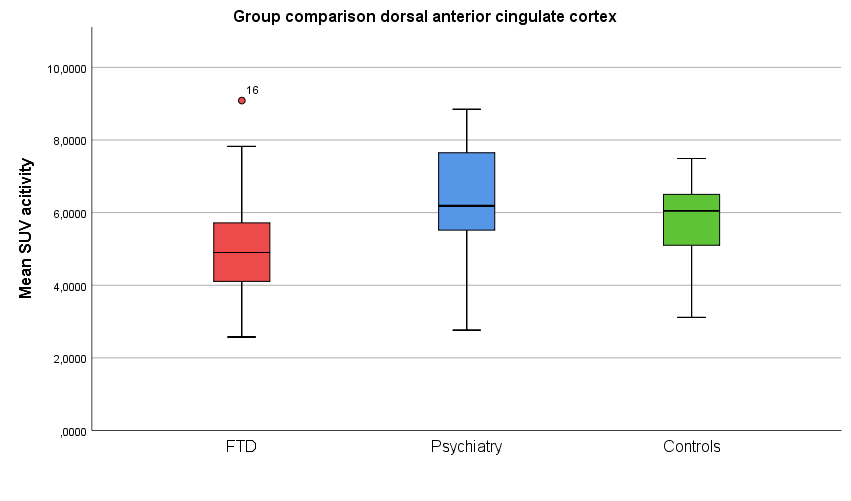

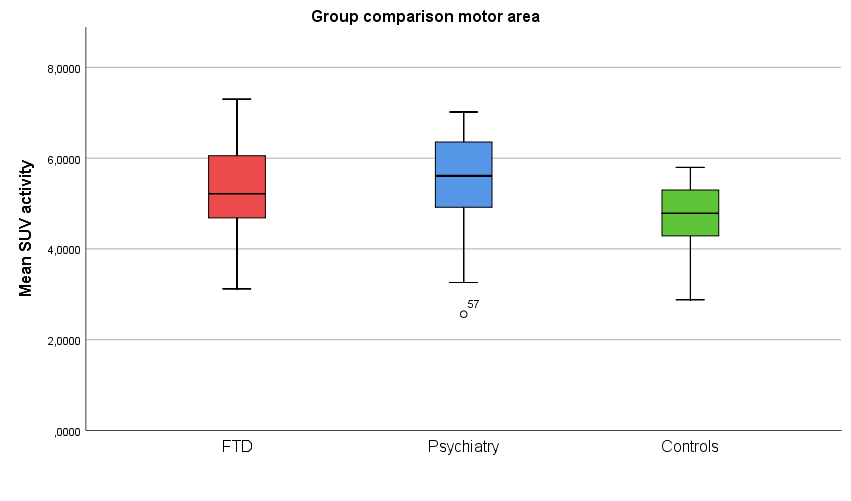

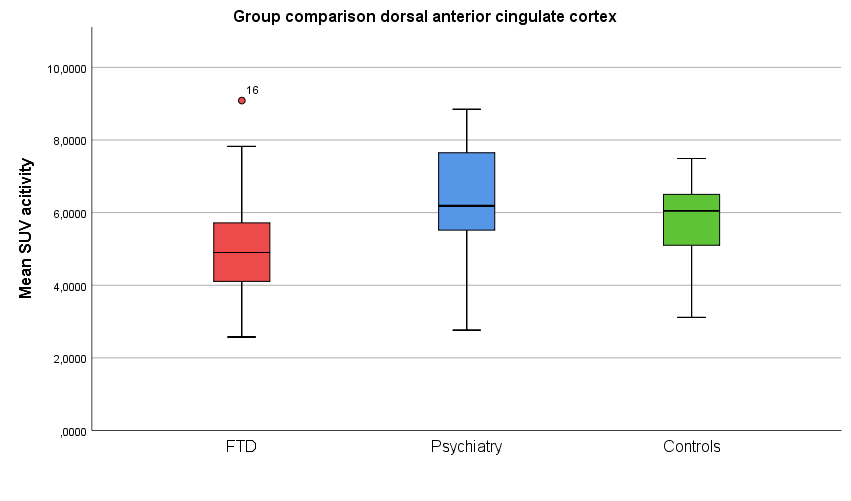
**Supplementary Fig. 1 Region of interest SUV activity across groups**

**Controls**

**BvFTD**

**A**

**PPD**

95% CI 5.2-6.4

95% CI 5.8-6.9

95% CI 4.5-5.6


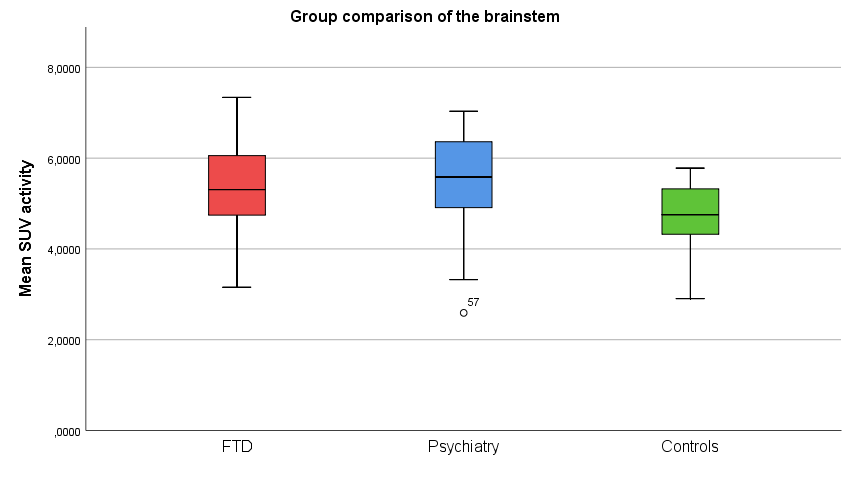


**B**

95% CI 4.3-5.1

95% CI 5.1-5.8

95% CI 4.9-5.7


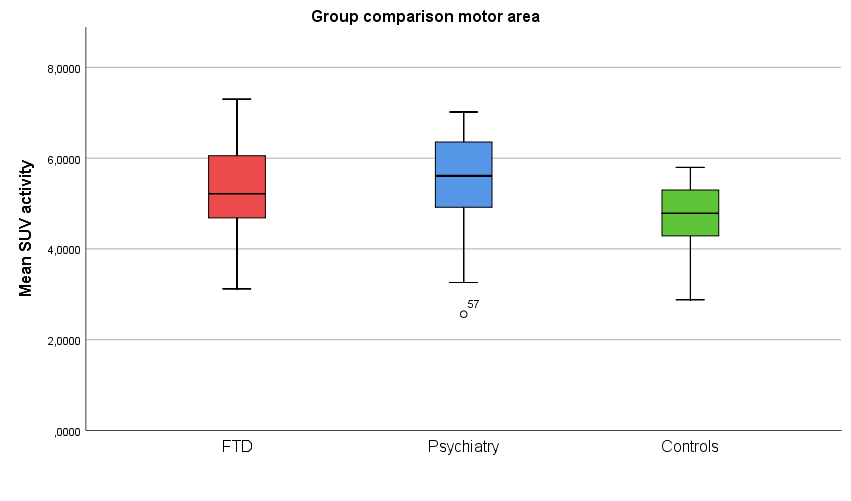


**C**

95% CI 4.3-5.1

95% CI 5.1-5.8

95% CI 4.9-5.7

Abbreviations: bvFTD = behavioral variant of frontotemporal dementia; PPD = primary psychiatric disorders. Data represent the regions of interest (ROI) of the most significant aberrant brain areas derived from the main analysis, of which the mean SUV_BW_ activity was extracted of each subject-scan. Boxplots show the group differences of the mean SUV_BW_ activity without using proportional scaling (raw data) of
**A) Dorsal anterior cingulate cortex B) Brainstem C) Motor cortex.**

**Exploratory analysis**
Exploratory whole brain analysis (with conservative threshold, i.e. FWE) was performed to investigate metabolism beyond the FTD mask (Supplementary materials Figure 2, Table 1) to reduce the risk of false negatives and because hypometabolism may occur in other brain regions.


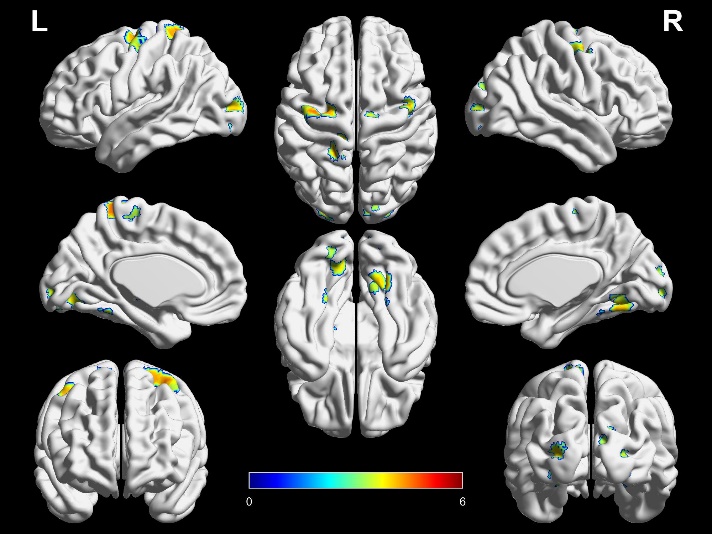

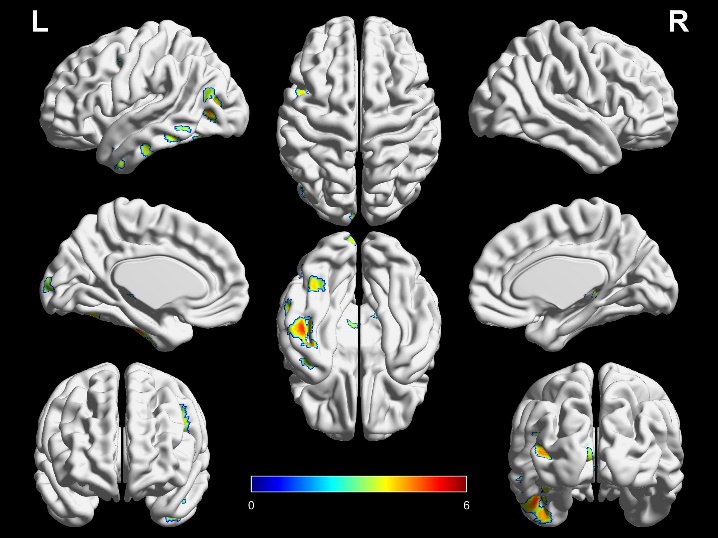

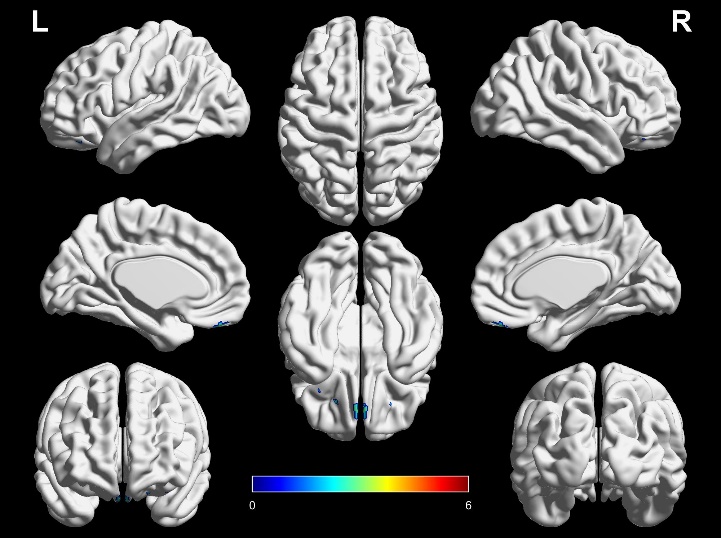

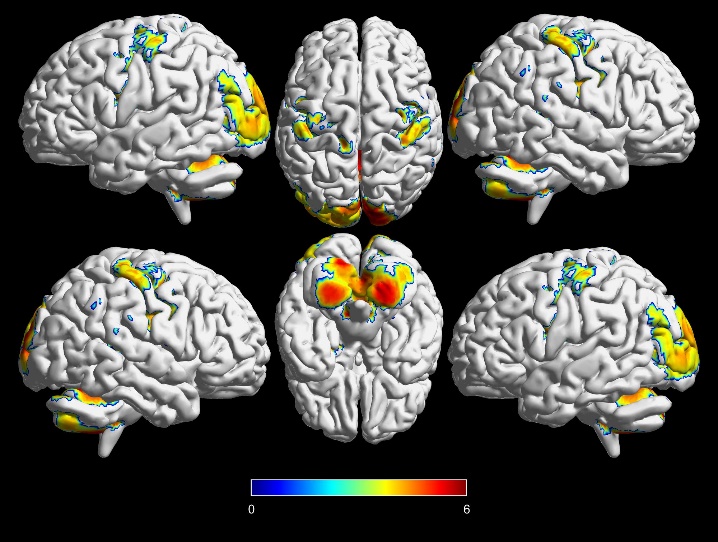

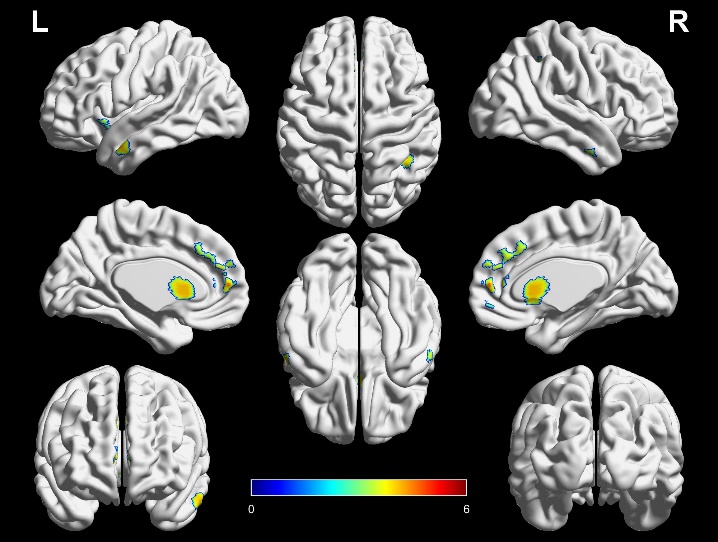

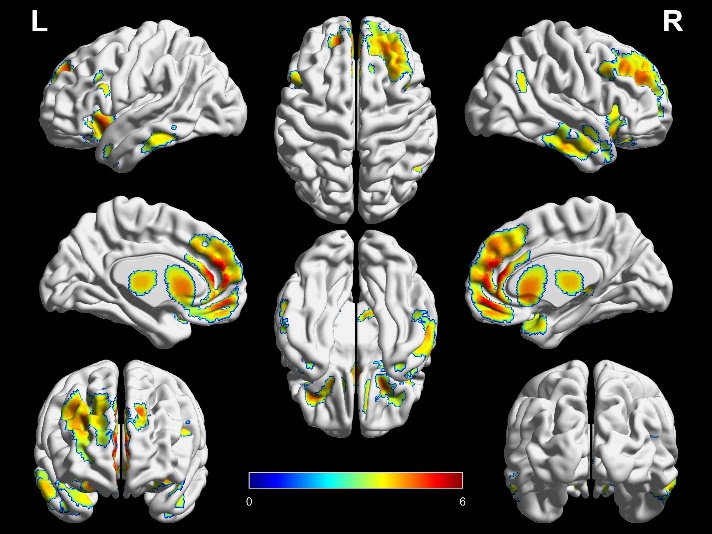


PPD > controls

**Supplementary materials Fig . 2 Functional brain abnormalities of FTD and PPD compared to controls – whole brain analysis**

Abbreviations: bvFTD = behavioral variant of frontotemporal dementia; PPD = primary psychiatric disorders Whole brain analysis; patterns of brain metabolism of FTD and PPD patients versus cognitively normal controls. Surface rendering of significant voxels from contrasts between FTD, PPD patients and controls are displayed at p < 0.001, extent threshold k  = 10. *thresholded at p<0.005 for visual purposes. Analysis of bvFTD versus controls was not adjusted for psychotropic drug use due to a small sample size. Significant clusters are displayed in table 4. Images were created with BrainNet Viewer[35]

PPD < bvFTD

bvFTD < PPD

bvFTD < controls

bvFTD > controls

PPD < controls*

**Supplementary materials Table 1. Brain regions with significant metabolic differences between bvFTD, PPD, controls (whole brain analysis)**

| Anatomical region | Laterality | MNI coordinates x y z | Cohen’s *d* | Cluster size in voxels |
| --- | --- | --- | --- | --- |
| **bvFTD < PPD**  Dorsal ACC Caudate  OFC Temporal  inferior temporal gyrus/temporal pole  fusiform gyrus  Thalamus  Amygdala Broca area  Angular gyrus  **PPD < bvFTD** Motor cortex  Cerebellum  Secondary visual cortex  **bvFTD < controls^c^** Dorsal ACC  Temporal cortex  Temporal pole  Inferior temporal gyrus  OFC  Angular gyrus  **bvFTD > controls^a^**  Brainstem, cerebellum  Thalamus  Motor cortex  Primary sensory cortex  Putamen  **PPD < controls** OFC **PPD > controls**  Visual associated cortex Temporal cortex  inferior temporal gyrus  fusiform gyrus  temporal pole  Thalamus  Brainstem  Motor cortex | bilateral bilateral left  right left bilateral  right left right  bilateral bilateral left  bilateral  left  right right right  bilateral right right  left  right  left  right  bilateral  left  left left  left  left  bilateral left | 0 44 16 8 14 -2 -38 20 -8  58 -18 -26 -58 -40 -16 4 -20 8 20 4 -24  -50 16 26 50 -58 26   -36 -12 62 16 -58 -20 -24 -94 6  0 48 6  -62 4 -22  62 -9 -34 -42 18 -8 40 -50 44  -2 -34 -12 18 -22 6 40 -10 58  -56 -2 20  -10 -36 70  -46 -19 51  30 -8 2  0 44 -25   -42 -74 6  -46 -24 -28 -36 -60 -20  -40 4 -40  16 -32 2  -2 -32 -8 -48 2 30 | 0.77 0.64 0.61  0.54 0.50 0.54 0.52 0.49  0.49  0.57 0.56 0.50  0.73  0.61  0.57 0.53 0.57  1.18 0.77 0.76  0.74  0.67  0.68  0.68  0.53  0.66  0.69  0.55  0.55  0.52 0.59 0.55 | 4726 1815 710  566 88 426 351 81 59  224 2033 250  513  95  60 33 13  81047 245 642  300  407  192  485  46  124  114  35  17  24 125 53 |

Abbreviations: ACC= anterior cingulate cortex; dlPFC = dorsolateral-prefrontal cortex; dPCC = dorsal posterior-cingulate cortex, OFC = orbitofrontal cortex; MNI = Montreal Neurological Institute; PFC = Prefrontal cortex; SMA = supplementary motor area. All results are adjusted for age, sex, psychotropic drug use and type of scanner. Significance was set at P<.001, cluster size *k* > 10, FWE-uncorrected. The Cohen’s *d* was calculated based upon the sample size and SPM-derived T values, while using the following formula: Cohen’s *d* = t/[N]^1/2^. Interpretation of the effect size of Cohen’s *d* are as follows: 0.2 = small effect. 0.5 = moderate effect. 0.8 = large effect. ^a^adjusted for age, sex and type of scanner (not for psychotropic drug use due to smaller sample size).

**Brain metabolism associated with behavioral symptoms, social cognition and cognitive functioning – per diagnostic group**

In bvFTD (n=11), lower brain metabolism in the bilateral temporal cortices (right medial temporal gyrus p<0.001 and p<0.05_FWE_, left superior temporal gyrus, right fusiform gyrus), left Broca area, left OFC, bilateral motor area and right insula (p<0.001) was associated with worse facial emotion recognition. In PPD (n=18), there was no association between brain metabolism and facial emotion recognition.

In bvFTD (n=14), lower brain metabolism in the right Broca area, bilateral dorsolateral and anterior PFC (p<0.001 and p<0.05_FWE_) and left motor area (p<0.001) was associated with stronger compulsive behavior, whereas in PPD (n=27) only the bilateral motor area was associated with stronger compulsive behavior (p<0.001).

Only in bvFTD (n=25), lower metabolism in the right dlPFC (p<0.001 and p<0.05_FWE_) and bilateral motor area was associated with worse letter fluency/executive functioning (Letter-DAT test) (p<0.001). In PPD (n=26) no associations were found between brain metabolism and letter fluency/executive functioning.

In bvFTD and PPD, there was no association between brain metabolism and depressive symptoms (MADRS), mental flexibility (TMTB), semantic fluency (Fluency60), executive functioning (Stroop 3) or color-word interference.

**Table 2. Voxel-wise contrasts of brain metabolism within the FTD mask in sporadic bvFTD, PPD and controls**

| Anatomical region | Laterality | MNI coordinates x y z | Cohen’s *d* | Cluster size in voxels |
| --- | --- | --- | --- | --- |
| **Sporadic bvFTD < PPD**  Dorsal ACC^#^  Subgenual ACC  dlPFC Frontal eyefields Temporal cortex  Medial temporal gyrus^#^  Temporal pole^#^  OFC  **Sporadic bvFTD > PPD** Motor cortex (pre-motor/SMA)  **Sporadic bvFTD < controls^c^**  Dorsal ACC OFC dlPFC Frontal eyefields Temporal cortex  Medial temporal gyrus^#^  Temporal pole  Fusiform gyrus Caudate  **Sporadic bvFTD > controls^a^** Motor cortex (pre-motor/SMA)  **PPD > controls^b^** Temporal cortex  Inferior temporal gyrus  Fusiform gyrus  Temporal pole  Thalamus | left  left  left right  right  left  left  left right  bilateral left  left right  right  left  left  right  left right  left left  left right | -2 46 12  -4 20 -16  -12 46 38  34 24 46  58 -2 -20  -46 10 -32  -38 28 -12  -32 -12 63  44 -6 54  2 44 4^&^  -38 28 -12  -12 46 38 34 24 46  58 -2 -20  -46 10 -32  -58 -40 -18  6 18 -2  -32 -12 64 44 -6 54   -46 -24 -28  -56 -42 -16 -40 4 -40 18 -29 4 | 0.69  0.47  0.49  0.57  0.72  0.70 0.59  0.65  0.58  0.60  0.76  0.62 0.55  0.92  0.87  0.76  0.59  0.83 0.74  0.69  0.53 0.50 0.51 | 1799  33  33  237  1646  954  299  171  33  49 321  351 278  3726  569  351  11  198 36   106  25  17 22 |

Abbreviations: ACC= anterior cingulate cortex; dlPFC = dorsolateral-prefrontal cortex; OFC = orbitofrontal cortex; MNI = Montreal Neurological Institute; PFC = Prefrontal cortex; SMA = supplementary motor area. Significance set at P < .001, cluster size *k* > 10, FWE-uncorrected. Results are adjusted for age, sex, psychotropic drug use and type of scanner. ^a^adjusted for age, sex, type of scanner (not for psychotropic drug use due to smaller sample size). The Cohen’s *d* was calculated based upon the sample size and SPM-derived T values, while using the following formula: Cohen’s *d* = t/[N]^1/2^. Interpretation of the effect size of Cohen’s *d* are as follows: 0.2 = small effect. 0.5 = moderate effect. 0.8 = large effect.. ^&^MNI coordinates of right side are displayed. # < 0.05_FWE_

**References**

35. Xia, M., J. Wang, and Y. He, BrainNet Viewer: a network visualization tool for human brain connectomics. PloS one, 2013. 8(7): p. e68910.
